# Controllable Preparation of Millimeter‐Scale In‐Plane Charged Domain Wall in a Lead‐Free Ferroelectric Film

**DOI:** 10.1002/advs.202505069

**Published:** 2025-06-30

**Authors:** Fang Liu, Mei‐Xiong Zhu, Jia‐Qi Liu, Li‐Xin Yang, Yu‐Jia Wang, Yin‐Lian Zhu, Xiu‐Liang Ma, Yun‐Long Tang

**Affiliations:** ^1^ Shenyang National Laboratory for Materials Science Institute of Metal Research Chinese Academy of Sciences Wenhua Road 72 Shenyang 110016 China; ^2^ School of Materials Science and Engineering University of Science and Technology of China Wenhua Road 72 Shenyang 110016 China; ^3^ Bay Area Center for Electron Microscopy Songshan Lake Materials Laboratory Dongguan 523808 China; ^4^ School of Materials Science and Engineering Hunan University of Science and Technology Xiangtan 411201 China; ^5^ Quantum Science Center of Guangdong‐HongKong‐Macau Greater Bay Area Shenzhen 518000 China; ^6^ Institute of Physics Chinese Academy of Sciences Beijing 100190 China

**Keywords:** atomic scale, charged domain wall, in‐plane charged antiphase boundary, lead‐free, pulsed laser deposition

## Abstract

Ferroelectric domain walls are emerging as active components in nanoelectronics, offering a transformative paradigm for non‐volatile memory and logic technologies. In particular, in‐plane charged domain walls stand out for their potential to support unique functionalities, such as quantum confinement and tunneling effects, unlocking new possibilities for advanced device applications. Here, a facile approach is developed for fabricating large‐scale continuous in‐plane charged domain walls in Na_0.5_Bi_0.5_TiO_3_ ferroelectric films through thermally optimized growth control. Using Differential Phase Contrast Scanning Transmission Electron Microscopy, an electric field fluctuation is identified across the charged domain wall. This fluctuation is accompanied by oxygen octahedron distortions, which allow the lattice to redistribute charges, as confirmed by integrated Differential Phase Contrast analyses. Additionally, Electron Energy Loss Spectroscopy reveals variations in titanium oxidation states across the domain wall, which compensates for the charge accumulation and enhances the stability of the charged domain wall. This work presents a wafer‐scale fabrication strategy for in‐plane charged domain walls and provides atomic‐scale insights into their stabilization mechanisms, offering a foundation for their integration into domain‐wall‐based nanodevices and on‐chip applications.

## Graphical abstract

This study presents an effective method for fabricating millimeter‐scale continuous in‐plane head‐to‐head charged domain walls by controlling the growth of the Na_0.5_Bi_0.5_TiO_3_ film. The formation of antiphase boundaries plays a crucial role in stabilizing the charged domain walls. Atomic‐scale insights reveal that the dynamic coupling among polarization, octahedron distortions, and charge determines their stability.

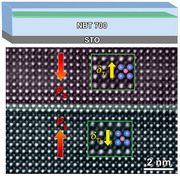

## 1 Introduction

Oxide 2D interfaces have emerged as a transformative platform for next‐generation device applications, offering unparalleled opportunities to tailor material properties at the atomic scale.^[^ ^1^, ^2^ ^]^ These interfaces, characterized by their high tunability and multifunctional capabilities, have driven the exploration of emergent phenomena such as interfacial superconductivity,^[^ ^3^, ^4^ ^]^ topological states,^[^ ^5^ ^]^ and exotic magnetism.^[^ ^6^ ^]^ Their potential to manipulate electronic, ionic, and spin‐related properties places them at the forefront of advanced materials research. Moreover, the scalability and compatibility of these interfaces present a viable solution for addressing challenges in promoting miniaturization and improving energy efficiency in modern electronic systems.^[^ ^7^ ^]^ Among these interfaces, ferroelectric domain walls have garnered significant attention as unique functional interfaces,^[^ ^7^, ^8^, ^9^, ^10^ ^]^ including photovoltages,^[^ ^11^ ^]^ conductivity,^[^ ^12^, ^13^, ^14^ ^]^ electromechanical properties,^[^ ^15^ ^]^ optical response,^[^ ^16^ ^]^ and magnetoelectric coupling.^[^ ^17^ ^]^ Such multifunctionality establishes ferroelectric domain walls as critical players in the exploration of interfacial phenomena, paving the way for advanced materials and devices.

Ferroelectric domain walls, naturally forming in materials with spontaneous polarization, act as boundaries that separate chemically uniform regions with distinct polarization orientations.^[^ ^18^, ^19^, ^20^, ^21^, ^22^ ^]^ They are classified as neutral (head‐to‐tail or antiparallel) and charged (head‐to‐head or tail‐to‐tail) types based on the presence or absence of bound charges.^[^ ^23^, ^24^ ^]^ Additionally, they are classified as out‐of‐plane or in‐plane walls based on their orientation relative to the film surface. In ferroelectric thin films with out‐of‐plane polarization, neutral out‐of‐plane domain walls are typically dominant, minimizing both the domain‐wall energy density and the domain‐wall area. In contrast, in‐plane charged domain walls (CDWs) are rarely observed due to their high electrostatic energy and the extensive lateral area not limited by film thickness.^[^ ^25^, ^26^ ^]^ Despite this, in‐plane CDWs exhibit unique functional properties distinct from those of out‐of‐plane CDWs, particularly in controlling electronic transport phenomena.^[^ ^25^ ^]^ Notably, the polarization switching of in‐plane CDWs has also been proposed to control tunneling conductance in ferroelectric tunnel junctions, where the induced quantum confinement can lead to resonant tunneling and electroresistance effects.^[^ ^27^ ^]^ These emerging functionalities highlight the significance of in‐plane CDWs for next‐generation nanoelectronic and neuromorphic applications. On the other hand, previous studies have demonstrated that domain walls can be pinned by various defects such as vacancies,^[^ ^24^ ^]^ dislocations,^[^ ^28^ ^]^ phase boundaries,^[^ ^29^, ^30^, ^31^ ^]^ and ferroelastic domains.^[^ ^32^ ^]^ Among these defects, antiphase boundary (APB), characterized by a half‐unit‐cell shift in registry relative to adjacent regions, has attracted significant attention due to its ability to locally alter polarization and affect device performance.^[^ ^33^ ^]^ Therefore, controlling the formation of the APB is crucial for exploring new functionalities and enhancing device performance. However, integrating in‐plane charged APB into ferroelectric films remains challenging, requiring feasible fabrication techniques and deeper insights to unlock their potential in ferroelectric device engineering.

In this report, Na_0.5_Bi_0.5_TiO_3_ (NBT) serves as a model system due to its prominence in the exploration of lead‐free ferroelectric materials.^[^ ^34^, ^35^, ^36^ ^]^ NBT exhibits a distorted perovskite structure with notable chemical disorder, cation displacements, and octahedron tilt, alongside a maximum relative permittivity of *ε*_r_≈3000 at ≈320 °C (*T*_max_).^[^ ^37^ ^]^ This distorted structure ensures stable high‐temperature permittivity, essential for ceramic capacitors, while supporting macroscopic ferroelectricity and phase adaptability. These characteristics make the material well‐suited for non‐volatile memory, electromechanical devices, and energy storage systems.^[^ ^38^, ^39^ ^]^ Moreover, NBT is highly sensitive to growth parameters, allowing for precise control of its microstructural characteristics. For instance, NBT can function as an ionic conductor, with the oxygen vacancy concentration via the charge compensation mechanism driven by bismuth loss at elevated growth temperatures.^[^ ^37^ ^]^ The precise microstructural manipulation of NBT further enables refined control over its ferroelectric and dielectric properties, which is crucial for advancing functional nanoelectronics devices.^[^ ^40^, ^41^ ^]^ Consequently, the development of high‐quality NBT epitaxial films with meticulously tailored microstructures offers a versatile platform for exploring novel functionalities and driving advancements in nanoelectronics.

Here, we demonstrate the controlled fabrication of spatially continuous in‐plane charged APB in the NBT thin film through optimizing growth conditions. NBT thin films were deposited on (001)‐oriented Nb:SrTiO_3_ (STO) by pulsed laser deposition (PLD) at growth temperatures ranging from 600 to 800 °C, with 700 °C producing large‐scale continuous in‐plane charged APB. High‐angle annular dark‐field scanning transmission electron microscopy (HAADF‐STEM) and atomic‐resolution energy‐dispersive X‐ray spectroscopy (EDS) elucidate the detailed atomic configurations at the APB, while Differential Phase Contrast (DPC) imaging maps the electric field distribution across the APB. Through quantitative analysis of atomic column positions, we identified an atomically sharp head‐to‐head polarization reversal associated with the in‐plane CDWs. Integrated Differential Phase Contrast (iDPC)‐STEM and electron energy loss spectroscopy (EELS) further reveal the stabilizing mechanism of the CDWs, including octahedron distortion and changes in the valence states of Ti, which alleviate the instability caused by charge asymmetry. This work introduces an effective approach for fabricating large‐scale in‐plane CDWs through growth modulation and offers atomic‐scale structural insights, paving the way for leveraging these unique features in next‐generation nanoelectronics and memory devices.

## 2 Results and Discussion

**Figure 1:**
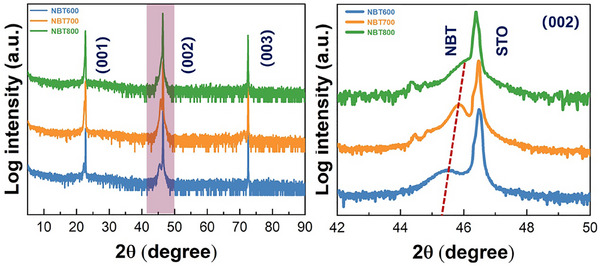
Crystal structure characterization of NBT films. a) XRD *θ* − 2*θ* patterns of the NBT thin films. b) Local *θ* − 2*θ*  XRD scans of NBT films.

**Figure 2:**
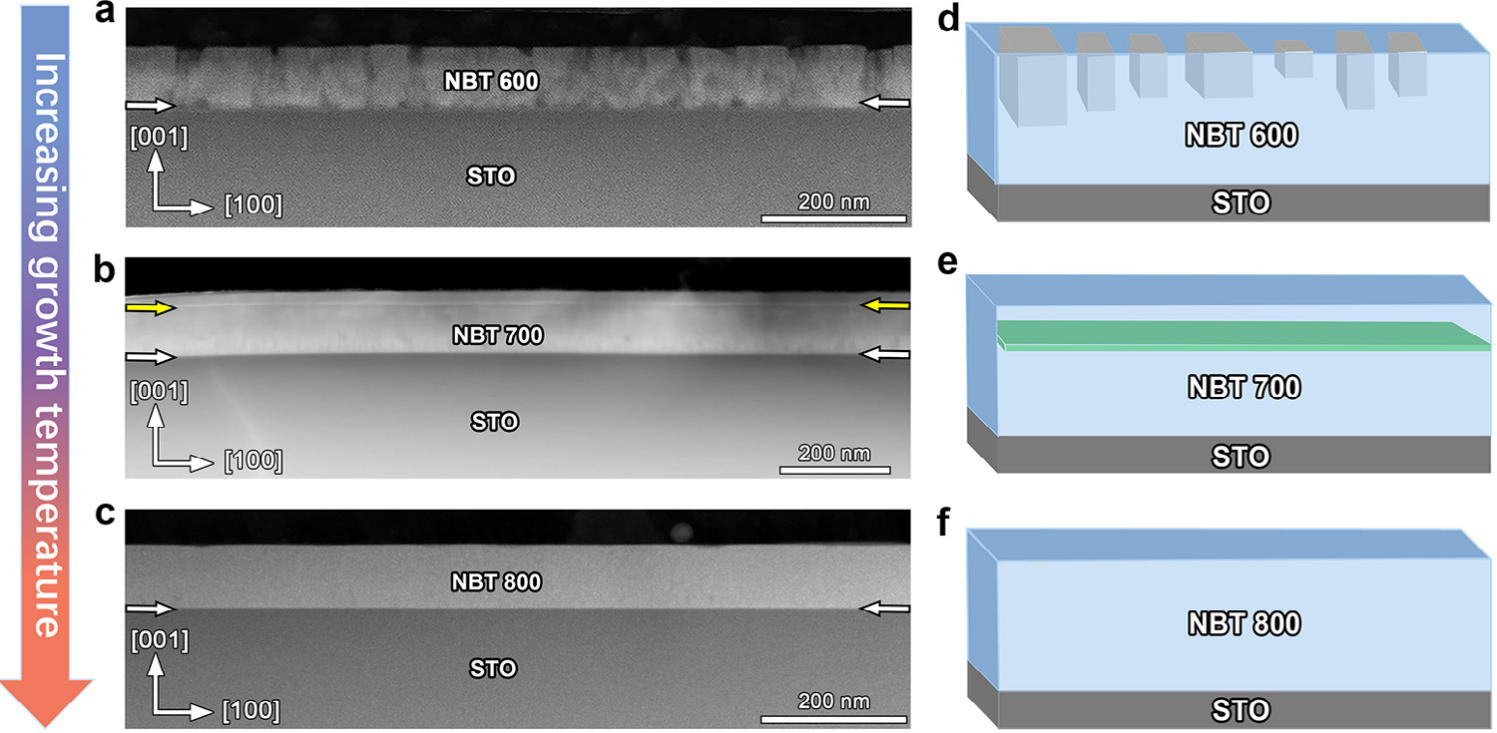
Temperature‐dependent microstructural evolution of NBT films on STO (001). Cross‐sectional HAADF‐STEM images of NBT films at growth temperatures of a) 600 °C, b) 700 °C, and c) 800 °C. d–f) Schematic models illustrating structural evolution corresponding to the growth temperatures shown in (a–c).

NBT films were deposited on (001)‐oriented STO substrates using the PLD technique at growth temperatures of 600, 700, and 800 °C, denoted as NBT600, NBT700, and NBT800, respectively. The growth temperature plays a critical role in shaping the microstructural evolution of thin films, particularly in systems containing volatile elements, through its impact on atomic mobility, defect formation, and crystallinity.^[^ ^33^ ^]^ **Figure** **1**a shows high‐resolution X‐ray diffraction (HRXRD) *θ* − 2*θ* scans of NBT films grown at various temperatures. The broad 5°−90° scan reveals indexed (00*l*) peaks, with no additional peaks observed, indicating a degree of crystalline ordering. This is further confirmed by the local HRXRD patterns near the STO (002) peak, shown in Figure 1b, in the 42°–50° range. To further investigate the microstructural features of the films, HAADF‐STEM images were performed. **Figure** **2**a–c display pronounced temperature‐driven structural variations. Three NBT films grown under different conditions show very different surface morphologies and structural variations. Figure 2d–f present schematic growth models that capture microstructural evolution at different growth temperatures. The NBT600 film (Figure 2a,d) displays a rough surface and columnar structures near the surface. These features probably arise from insufficient atomic mobility at low temperatures, which limits atom diffusion. The NBT700 film (Figure 2b,e) exhibits a unique intermediate state, highlighted by yellow arrows, likely associated with planar irregularities such as stacking faults or some twin boundaries. These features appear to result from a fine balance between surface diffusion and lattice relaxation at the moderate growth temperature, leading to a transitional microstructure that connects the characteristics observed at higher and lower growth temperatures. In contrast, the NBT800 film (Figure 2c,f) exhibits a smooth, uniform surface with minimal structural distortions. This is generally associated with increased atomic mobility at elevated temperatures, facilitating atom diffusion and promoting the formation of a smooth surface. Among the three samples, the NBT700 film stands out due to its prominent in‐plane structural feature, offering a promising platform for investigating defect‐mediated functionalities. Accordingly, the subsequent discussion will focus on the exceptional structural characteristics and potential applications of NBT700 film.

**Figure 3:**
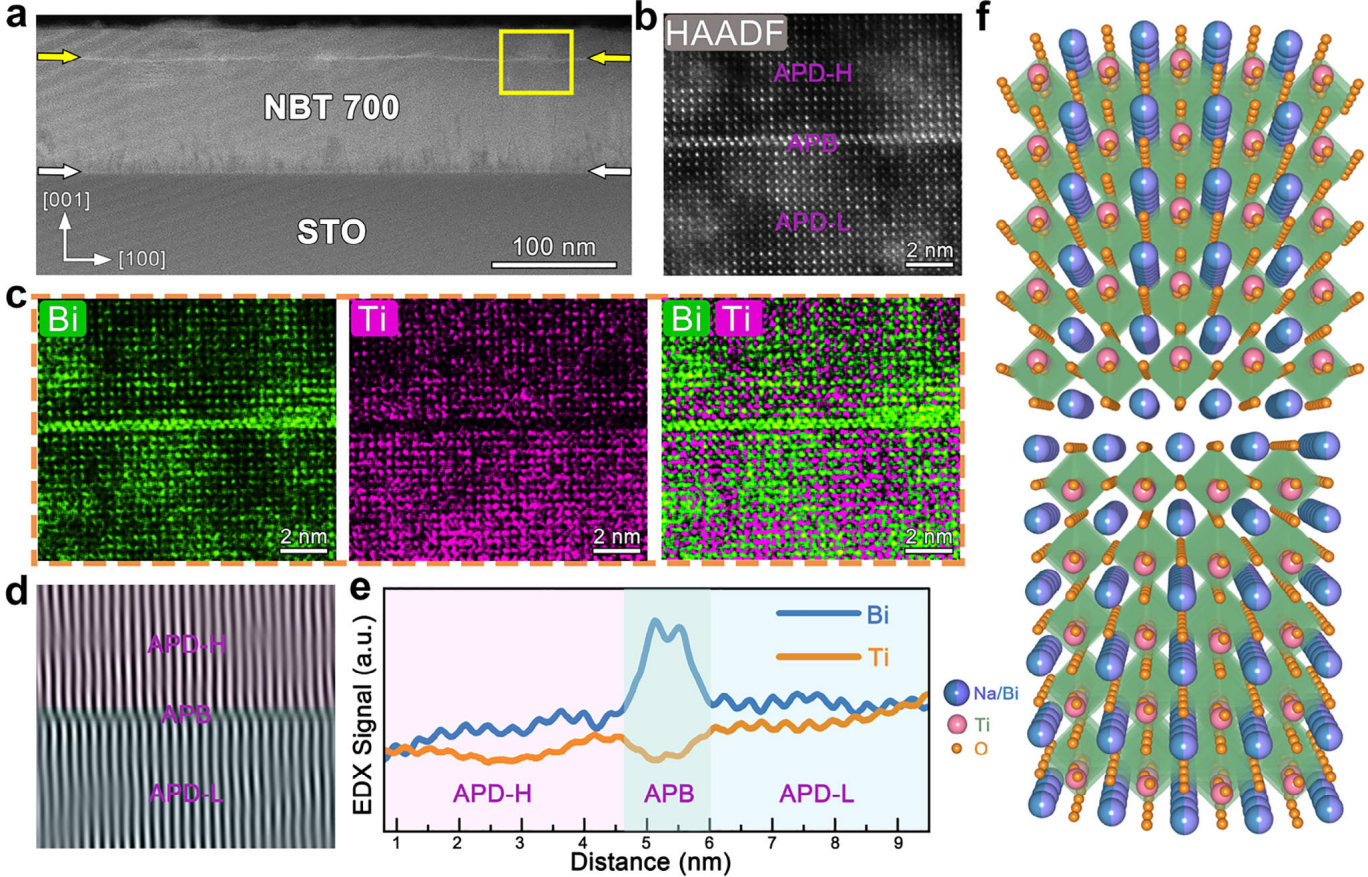
Atomic resolved structure and composition analysis of the APB. a) HAADF‐STEM image of the NBT700 film projected along the [010] zone axis. b) The high‐magnification HAADF‐STEM image of the yellow rectangle in (a). c) Atomically resolved EDS mapping determining the atomic configuration in the APB corresponding to (b). d) The inverse fast Fourier‐filtered pattern of (b). e) Differences between Bi and Ti elements with line profiles across the APB and APD. f) A sketch of the atomic structure of the APB. Blue/purple, green, and orange balls represent Bi/Na, Ti, and O, respectively.

To investigate the in‐plane large‐scale structural feature discussed above at the atomic level, we performed detailed analyses using HAADF‐STEM and EDS mapping (**Figure** **3**). As shown in Figure 3a, the in‐plane defect (highlighted by yellow arrows) exhibits brighter contrast, suggesting the region is probably enriched with heavier elements. Atomic‐scale HAADF‐STEM imaging (Figure 3b) further identifies a half‐unit‐cell shift beside two adjacent regions, which possibly corresponds to APB in NBT700 thin films. The film thickness is ≈115 nm, with APB notably emerging at a depth of ≈20 nm from the top surface. The APB near the surface may result from the increased sensitivity of the surface to local chemical and electrostatic perturbations, which can induce polarization discontinuities.^[^ ^42^ ^]^ For clarity, we designate the lower and higher regions across the APB as APD‐L and APD‐H, respectively, emphasizing the need to assess their effect on the material's properties. Normal strain maps in the out‐of‐plane and in‐plane direction derived from Geometric Phase Analysis (GPA) (Figure S1, Supporting Information) reveal significant lattice distortions at the APB. Atomic‐scale EDS mapping (Figure 3c) and the corresponding line profile (Figure 3e) reveal significant Bi enrichment and Ti absence at the APB. These compositional variations indicate that localized chemical variation at the APB may contribute to its distinctive electronic properties. Furthermore, the inverse fast Fourier transform (IFFT) of the interface is shown in Figure 3d, providing direct evidence of the characteristic half‐unit‐cell shift in atomic registry relative to adjacent regions. The corresponding microstructural schematic (Figure 3f) further illustrates the in‐plane APB, emphasizing the unique atomic arrangement. Furthermore, we investigated the ferroelectric response of the NBT700 film by PFM. Figure S2a (Supporting Information) presents the out‐of‐plane PFM amplitude image of locally written domains that display clear boundaries. The corresponding phase image in Figure S2b (Supporting Information) exhibits a 180° contrast between oppositely poled regions. This contrast can be overwritten by applying an opposite electric field, as depicted in Figure S2c (Supporting Information). In addition, the NBT700 film exhibits a distinctive butterfly‐shaped amplitude curve and a 180° phase hysteresis loop in Figure S2d (Supporting Information), further confirming local ferroelectric switching behavior. Thus, despite the structural distortions, the NBT700 film retains ferroelectric properties and remains switchable under an applied external field, suggesting the stability and functionality of its ferroelectric phase.^[^ ^43^ ^]^ Such APBs are not merely structural irregularities but may act as sites for local polarization or charge trapping, potentially influencing macroscopic behaviors such as conductivity, stability, and mechanical response.^[^ ^44^ ^]^ Understanding these distinctive features is critical for unraveling the interplay between localized defect chemistry and overall material performance.

**Figure 4:**
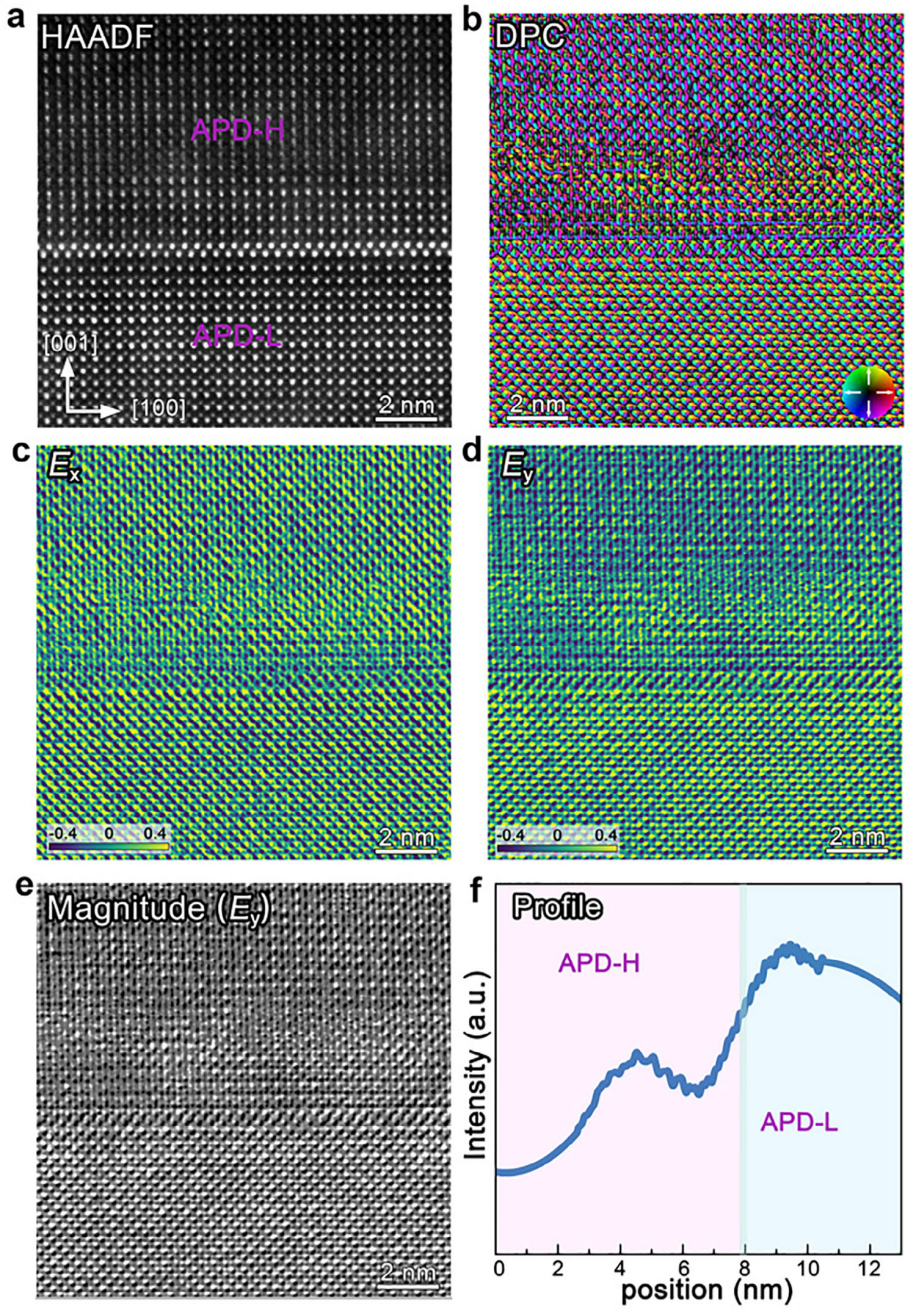
DPC‐STEM measurements of electric field distribution across the APB. a) HAADF‐STEM of the NBT700 thin film. b) Vector map of the DPC signal, indicating the direction and magnitude of the phase shift. c) *E*_x_ map of the DPC signal, representing the electric field component along the x‐axis. d) *E*_y_ map of the DPC signal, representing the electric field component along the y‐axis. e) Magnitude of the *E*_y_ component, showing intensity variations across the APB. f) The electrical intensity along the [001] direction, displaying an obvious accumulation of polarization charges in APD‐L.

To investigate the influence of atomic‐scale chemical variation near the APB on polarization behavior, the DPC‐STEM imaging technique was employed to map the local electric field distribution within the NBT700 film. A cross‐sectional image of the NBT700 film was obtained using HAADF‐STEM, as shown in **Figure** **4**a. The corresponding DPC signal, derived from intensity differences across the four detector segments (Figure S3, Supporting Information) and normalized to the total intensity of the Bright Field disk during data processing, is displayed in the vector plot of Figure 4b, where the phase is represented in color and magnitude in saturation. In this DPC image, the vectors represent the projected local electric field within the film. The diverse color scales in the DPC image possibly reveal in‐plane CDWs linked to the APB in the NBT700 film. The *E*_x_ (electric field component along the x‐axis) and *E*_y_ (electric field component along the y‐axis) components were extracted by calculating the difference between opposing quadrant signals on the DPC detector in Figure 4c,d, respectively. As shown in the *E*_y_ map, a distinct color contrast appears across the APB, with APD‐H displaying a yellow tone and the APD‐L exhibiting a blue tone, indicating the opposite charge distribution. In contrast, the *E*_x_ map shows nearly consistent field directions, suggesting the uniform charge distribution across the APB. These distinct field patterns confirm that the APB is oppositely in‐plane charged, as visualized through the DPC signal color mapping. For further clarity, Figure 4e illustrates the magnitude of the *E*_y_ components, which excludes the phase information. In particular, the line profile of the *E*_y_ magnitude signal (Figure 4f) displays a marked enhancement in the APD‐L region, where the intensity surpasses that of the APD‐H region. This arises from the electric field intensity across the APB driven by variations in local electrostatic properties and suggests that the APB is probably a CDWs.^[^ ^45^ ^]^ Exploring the transport behavior through cross‐sectional conductive atomic force microscopy (*c*‐AFM) could provide insights into its potential for device applications.^[^ ^26^ ^]^ Nevertheless, its precise configuration as head‐to‐head (positive bound charge) or tail‐to‐tail (negative bound charge) needs further study. Accordingly, we will examine the structure of CDWs to investigate its potential electronic properties and behavior.

**Figure 5:**
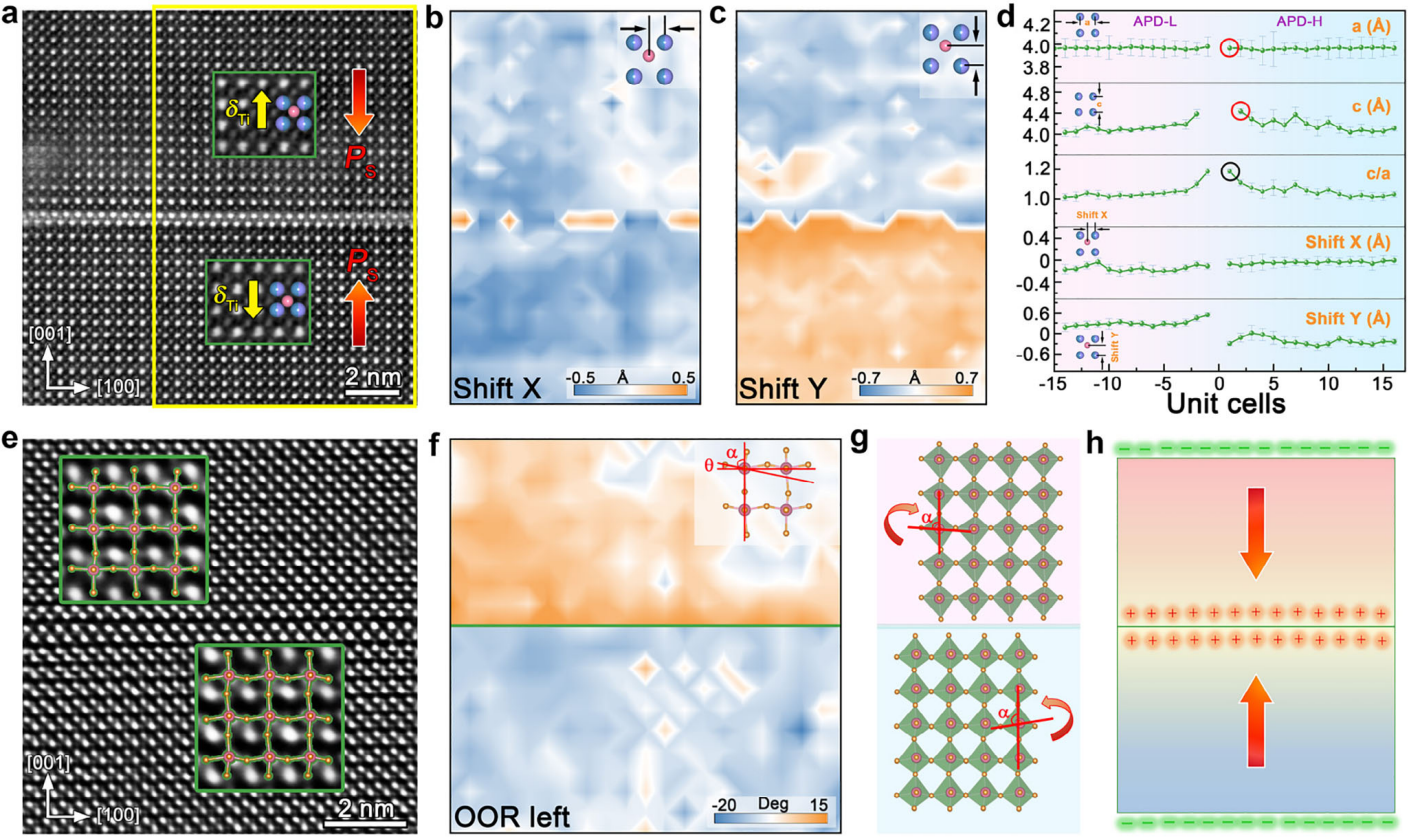
Polar features and oxygen octahedron distortion across the CDWs. a) HAADF‐STEM image across the in‐plane CDWs. The insets are enlarged images of each domain. The yellow arrows denote the Ti ionic displacements. The red arrows denote the polarization direction in each domain. b) Ti ionic displacements mapping of the yellow rectangle in (a) along the [100] direction. c) Displacement mapping along the [001] direction. Displacements toward the right in [100] or upward in [001] are defined as positive values. d) The extracted lattice parameters, tetragonality, and ionic displacements. The error bars denote the standard deviation of the corresponding dataset from each unit cell. e) The iDPC‐STEM image across the in‐plane CDWs. The green insets are a superposition of the atomic schematics with enlarged images of each domain. f) Variation of the rotation angle of the oxygen octahedron is defined as the angle (*θ*_left_ = 90° − *α*), defined by the angle between the Ti─O bond (Ti atom to the oxygen atom on the left) and the bond to an adjacent Ti atom. The green line denotes the CDWs. g) The model of octahedron distortion: APD‐H rotates clockwise, while APD‐L rotates counterclockwise. h) The model of CDWs.

Based on the investigation of local electric field distributions near the APB, the analysis is extended to explore the nature of CDWs in the NBT700 films and the mechanisms underlying their stability. High‐magnification HAADF‐STEM image clearly shows that the displacements of Ti ions are along opposite directions for the APD‐L and APD‐H regions (**Figure** **5**). The displacement vectors of Ti ions in each unit cell, as shown in Figure 5a, align in the opposite direction relative to the polarization direction of the NBT700 film, forming a head‐to‐head polar configuration. Figure 5b,c provides quantitative mapping of the Ti ion displacements along the in‐plane (shift X) and out‐of‐plane (shift Y) directions, respectively. Notably, shift X is uniformly on both sides of the CDWs. In contrast, shift Y undergoes an almost 180° inversion, signifying a complete reversal in displacements direction across the CDWs. Specifically, the maximum Ti ion displacements reach a value of ≈0.5 Å at the CDWs. By extracting atomic position data and calculating lattice parameters for each unit cell (red‐boxed region, Figure 5d), significant structural distortions have been identified. The out‐of‐plane lattice constants as large as ≈4.4 Å are observed near the APB, while the in‐plane lattice constants stabilize at ≈3.9 Å on both sides of the APB (red circle). This induces a marked increase in tetragonality, with values reaching as high as ≈1.2 (black circle), highlighting a significant structural change. The enhanced tetragonality at the domain wall may be attributed to the accumulation of positive charges at the head‐to‐head domain wall, which could create a localized electrostatic field. To minimize the electrostatic energy, the system undergoes structural elongation along the out‐of‐plane direction, redistributing the charge and broadening the domain wall region.

For perovskite oxide, properties are strongly influenced by oxygen octahedron rotation and distortion, which significantly affect electronic and structural behavior. To investigate the effect of the CDWs on the oxygen octahedron distortions, we performed iDPC‐STEM, which enables the simultaneous imaging of heavy elements (Bi and Ti) and light elements (O). High‐magnification iDPC‐STEM images clearly show that O ions displacements are along opposite directions for the APD‐L and APD‐H regions, further demonstrating the formation of a polarization arrangement with opposing directions, as shown in Figure 5e. At CDWs, the coupling effect between polarization charges and lattice structures significantly modulates local physical properties. Figure 5f shows the rotation angle of the oxygen octahedron (OOR‐left) mapping, which represents the rotation angle of the oxygen octahedron on the left (*θ*_left_ = 90° − *α*) and *θ*_left_ is defined by the deviation between the Ti─O bond (from Ti atom to the oxygen atom on the left) and the bond connecting the Ti atom to an adjacent Ti atom. Figure 5g illustrates the octahedron distortion model, with APD‐H rotating clockwise and APD‐L rotating counterclockwise. Meanwhile, it can be observed that the degree of octahedron distortion in APD‐L is more pronounced than that in APD‐H. We conducted multislice simulations using the experimental imaging parameters to verify the observed oxygen octahedral distortions. As shown in Figure S4 (Supporting Information), the octahedral rotation pattern remains consistent up to a tilting angle of ±8 mrad. Significant distortions only emerge at large tilts (>10 mrad), which is significantly beyond the experimental tilt uncertainty. This confirms that the experimentally observed distortions are intrinsic to the structure, not imaging artefacts. The electric field fluctuation near the CDWs spatially correlates with localized octahedron distortions in the lattice structure (Figure 4). To compensate for this electric field fluctuation, the lattice responds to the local stress field through octahedron distortions. This coupling effect indicates that CDWs are not merely a simple result of charge accumulation but are accompanied by complex structural adjustments. This highlights the critical role of charge‐lattice distortion interactions in regulating local electrical and structural properties.

**Figure 6:**
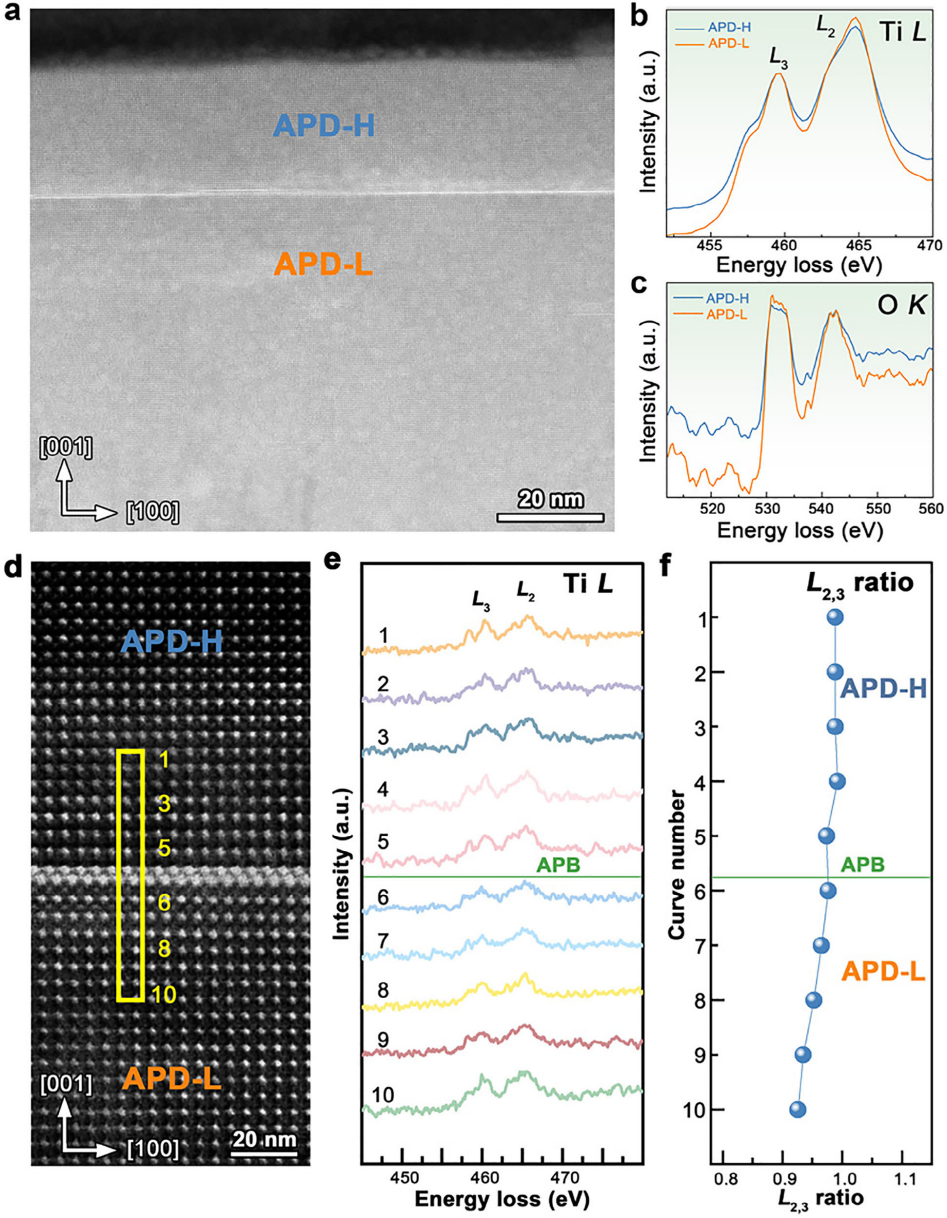
EELS measurements of oxidation state across the CDWs. a) HAADF‐STEM image showing the APD‐H and the APD‐L. b) The Ti *L*_2,3_ and c) The O *K* edges of the APD‐H (blue) and the APD‐L (orange), respectively. The APD‐L exhibits a smaller *L*_2,3_ ratio and a higher O *K* edge pre‐peak intensity. d) Atomic HAADF‐STEM image showing the APD‐H and the APD‐L. e) Ti *L*_2,3_ of the positions numbered from 1 to 10 corresponding to the EELS spectra. f) Ti *L*_2,3_ ratio of the APD‐H and the APD‐L.

To investigate the electronic properties of the CDWs, the fine structures of the Ti‐*L*_2,3_ and O‐*K* edges were further examined using EELS. **Figure** **6**a presents a HAADF‐STEM image containing the charged APB. These variations are clearly reflected in the Ti‐*L*_2,3_ and O‐*K* fine structures shown in Figure 6b,c, which display significant differences in their electronic structures. Specifically, after normalization, the APD‐L region exhibits a higher Ti‐*L*_2_ intensity and a more enhanced O‐*K* edge pre‐peak compared to APD‐H. The variations in the O‐*K* edge pre‐peak intensity and Ti‐*L*_2,3_ ratio provide valuable insights into the Ti valence state in these regions.^[^ ^46^ ^]^ The lower Ti‐*L*_2,3_ ratio observed in APD‐L suggests a higher oxidation state, as a higher oxidation state typically leads to a reduced Ti‐*L*_2,3_ ratio due to a diminished electron population in the 3*d* orbitals (Figure 6b). Furthermore, the pre‐peak in the O‐*K* edge spectrum corresponds to transitions to unoccupied Ti *3d*–O *2p* hybridized states. The higher pre‐peak intensity in APD‐L suggests an increased availability of unoccupied 3*d* states,^[^ ^46^ ^]^ further supporting this interpretation, suggesting a higher Ti valence state (Figure 6c). These observations are consistent with atomic‐scale EELS spectra (Figure 6d–f), which confirm that the Ti oxidation state is higher in the APD‐L region. This distinction in Ti oxidation states reflects the differing electronic environments in the APD‐H and APD‐L regions, driven by polarization asymmetry at the CDWs. The higher Ti valence state in APD‐L may act as a compensatory mechanism to mitigate the potential variations near the CDWs induced by localized charge distribution.

The large‐scale continuous in‐plane head‐to‐head CDWs in NBT films are stabilized via APB formation during epitaxial growth. Furthermore, we performed cross‐sectional HAADF‐STEM imaging at three randomly selected positions on a 5 mm × 10 mm sample. In all cases, laterally continuous APBs were observed across the full field of view (Figure S5, Supporting Information). Given the randomness of site selection, such consistency is statistically improbable unless the APBs extend uniformly across the sample. These results demonstrate that the APBs extend over millimeter scales and suggest the potential to reach wafer scale. In addition, this temperature‐driven growth strategy avoids the need for external perturbations, such as He‐ion implantation.^[^ ^26^ ^]^ The observed phenomenon reflects a complex interaction between thermodynamic factors, such as phase stability and surface energy minimization, and kinetic processes like atom mobility and nucleation rates during thin‐film deposition. By precisely regulating the temperature, APB‐mediated in‐plane CDWs patterning is achieved without the need for conventional post‐growth modifications. These results suggest that future investigations into the coupled evolution of defects during growth could offer deeper insight into the formation mechanism.

A critical discovery is the simultaneous stability of in‐plane CDWs and electrostatic incompatibility in head‐to‐head configurations (Figure 5h). The observed Bi segregation and Ti deficiency facilitate charge compensation at the APB, thereby contributing to its thermodynamic stabilization.^[^ ^47^ ^]^ In addition, the absence of a TiO_2_ layer at the APB disrupts the oxygen coordination environment, resulting in cooperative TiO_6_ octahedron distortion.^[^ ^33^ ^]^ The TiO_6_ octahedron distortion and charge density on the APD‐L side significantly exceed those on APD‐H, creating a spatially inhomogeneous screening environment (Figures 4 and 5). EELS analysis reveals a higher Ti valence state in APD‐L, directly linked to polarization‐induced electron redistribution near the CDWs (Figure 6). This valence shift may help regulate potential variations linked to charge accumulation, while lattice distortions could influence energy distributions to mediate electrostatic and structural effects. The dynamic coupling among polarization, octahedral distortions, and charge appears to play a role in stabilizing CDWs, differing from the carrier‐dominated screening generally observed.^[^ ^8^ ^]^ While the present work focuses on NBT, this approach can be extended to other bismuth‐based perovskites like BiFeO_3_. Such an extension may reveal new opportunities for engineering tunable ferroelectric interfaces. In addition, the position and configuration of CDWs may be influenced by growth conditions or external electric fields. Further investigation may offer new opportunities for controlled domain wall engineering. Notably, the micrometer‐scale lateral dimensions of continuous CDWs align with semiconductor lithography requirements, while their in‐plane orientation naturally accommodates planar integrated circuit designs. As the APB‐mediated patterning does not require post‐growth modulation, this feature could significantly reduce manufacturing complexity, offering new pathways for wafer‐scale high‐density ferroelectric memory fabrication. The atomic‐precision control of domain walls, combined with their scalability in lead‐free ferroelectrics, suggests potential for integration into electric devices such as non‐volatile memories or sensors. This work demonstrates a scalable fabrication of in‐plane CDWs and provides atomic‐scale insights into their stabilizing mechanisms, which paves the way to integrate CDWs at scales critical for on‐chip applications.

## 3 Conclusion

In summary, the controlled formation of large‐scale continuous in‐plane APB in NBT thin films was achieved by precisely regulating kinetic growth conditions. Our results demonstrate that the head‐to‐head CDWs can be effectively stabilized by the APB through growth temperature regulation. Detailed analysis shows that significant modifications in the oxygen octahedron configuration near the APB were observed, where polarization charge redistribution modifies the lattice structure that contributes to the stabilization of the CDWs. Additionally, variations in the oxidation states of Ti ions across the APB were identified as a critical compensatory mechanism to balance local charge imbalances, further enhancing domain wall stability. This approach offers a robust method for constructing large‐scale CDWs, enhancing understanding of atomic‐scale structure and stabilization mechanisms. The method combines practicality, adaptability, and scalability, enabling precise lateral patterning at the wafer scale.

## 4 Experimental Section

### Thin Film Deposition Details

The NBT thin films were deposited on STO (001) substrates by PLD with a Coherent ComPex PRO 201 KrF (λ = 248 nm) excimer laser. The stoichiometric NBT ceramic target was prepared by using the solid‐state sintering method. The substrate was pre‐heated to 850 °C for 10 min to clean the surface and then cooled down to 800, 700, and 600 °C before deposition, respectively. Then the NBT target was preputtered for 15 min to clean the surface. When growing the NBT films, the target‐substrate distance was 8 cm, the substrates were heated to deposition temperature, the repetition rate was 5 Hz, the oxygen partial pressure was 100 mTorr, and a laser energy density was 2.5 J cm^−2^ and then deposited. After growing the NBT films, the films were annealed at the deposition temperature for 5 min and then cooled down to 200 °C at a rate of 5 °C min^−1^ in an oxygen partial pressure of 200 Torr. Finally, the NBT films were furnace‐cooled slowly to room temperature.

### X‐Ray Diffraction

A crystal structure study was performed by X‐ray diffraction using a high‐resolution X‐ray diffractometer (Bruker, D8 Discover).

### STEM Sample Preparation

Cross‐sectional samples for STEM observation were prepared by slicing, gluing, mechanical grinding, dimpling, and finally ion milling by using the Gatan Precision Ion Polishing System 695. Before the ion milling, the samples were dimpled down to 10 µm by the Gatan 657 Dimple Grinder. In the initial stage of ion milling, the voltage was set at 4.5 kV, and the incident angle of the ion beam was 8°. Then, the voltage and incident angle were gradually reduced to 3.5 kV and 5°, respectively. Finally, the ion milling voltage was set at 0.1 kV for 5 min to reduce the amorphous layer produced by ion beam damage.

### Transmission Electron Microscopy

HAADF‐STEM images and elemental mapping were recorded using an aberration‐corrected scanning transmission electron microscope (Titan Cubed 60–300 kV microscope (FEI) fitted with a high‐brightness field‐emission gun (X‐FEG) and double Cs corrector from CEOS, and a monochromator operating at 300 kV). The atomic‐scale HAADF‐STEM images for acquiring the Ti displacement map are recorded by STEM Drift Corrected Frame Integration (DCFI). Each high‐resolution HAADF‐STEM image was acquired by adding up 20 original images with a dwell time of 100 ns. Image acquisition and processing were performed using the Velox software (FEI). Acquiring images in this way could reduce the influence of sample drift and scanning noises. The iDPC‐STEM imaging was performed using a four‐quadrant segmented detector (DF4 detector), recorded at 300 kV. EELS of O *K* and Ti *L*_2,3_ were acquired with an energy resolution of 0.8 eV (determined by the full‐width of the zero‐loss peak at half‐maximum). The DPC‐STEM imaging was performed using a four‐quadrant segmented detector (DF4 detector), recorded at 300 kV. The entrance aperture was selected to be 5 mm. Strain analyses were based on GPA using Gatan Digital Micrograph software. Simulated iDPC‐STEM images were obtained using abTEM, which is based on the multislice method.

### Peak Finding

To reduce the noise in the obtained images, the wiener filter was used for all atomic‐scale HAADF‐STEM images. By using a 2D Gaussian peak fitting in Matlab software, the atom positions could be accurately determined, thus making it possible to acquire information on the lattice constants, tetragonality, and ionic displacement.

### Ferroelectric Properties Characterization

PFM mappings were completed in a scanning probe microscope (Cypher S, Asylum Research) using the dual AC resonance tracking (DART) mode. Conductive silicon cantilevers with Ti/Ir coating (Asylum Research, Asyelec‐01‐R) were used for PFM imaging. PFM lithography poling was carried out with a DC switching voltage applied to the tip during scanning.

## Conflict of Interest

The authors declare no conflict of interest.

## Author Contributions

Y.‐L.T., Y.‐L.Z., and X.‐L.M. conceived and supervised the project. Y.‐L.T. and F.L. designed the experiments. F.L. conducted thin‐film growth. F.L., J.‐Q.L. and L.‐X.Y. conducted STEM observations. F.L., M.‐X.Z and Y.‐J.W. contributed to the data analysis. All authors participated in preparing the manuscript.

## Supporting information



Supporting Information

## Data Availability

The data that support the findings of this study are available from the corresponding author upon reasonable request.
