# Supplementary material for: Controllable Preparation of Millimeter‐Scale In‐Plane Charged Domain Wall in a Lead‐Free Ferroelectric Film
Source: Adv Sci (Weinh). 2025 Jun 30;12(34):e05069. doi: 10.1002/advs.202505069 (PMC12442660; doi:10.1002/advs.202505069)
Supplement: Supplementary file 1 — Supporting Information [file ADVS-12-e05069-s001.docx]

Supplementary Materials for

**Controllable Preparation of Millimeter-scale In-plane Charged Domain Wall in a Lead-free Ferroelectric Film**

Fang Liu^a,b^, Mei-Xiong Zhu^a,b^, Jia-Qi Liu^a^, Li-Xin Yang^a^, Yu-Jia Wang^a,b^, Yin-Lian Zhu^c,d^, Xiu-Liang Ma^c,e,f^, Yun-Long Tang^a,b^*

Correspondence to: Yun-Long Tang, yltang@imr.ac.cn


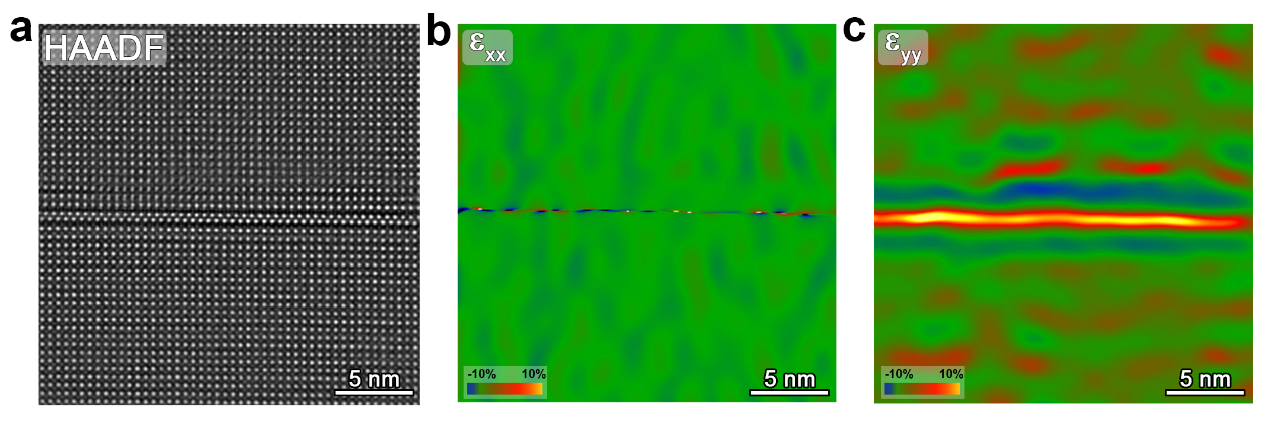
Figure S1. GPA analysis of NBT film. (a) Cross-sectional HAADF-STEM image of the NBT film. (b) and (c) Corresponding in-plane (*ε*_xx_) and out-of-plane (*ε*_yy_) strain maps, respectively.


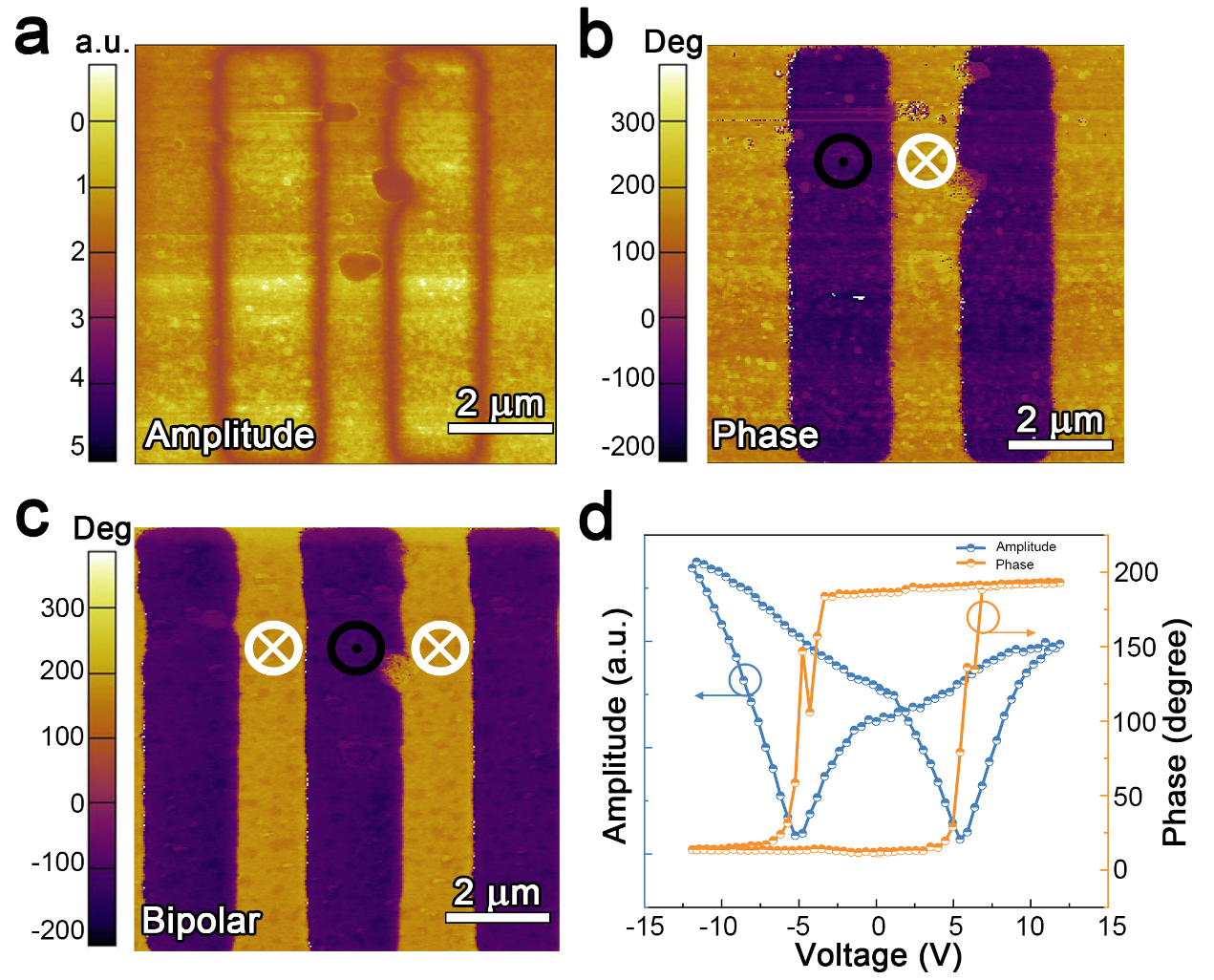
Figure S2. Characterization of ferroelectric properties at room temperature. (a-b) The PFM amplitude and phase mappings of electrically pre-poled domains. (c) The PFM phase mappings applied the electric field opposite to (b). (d) Amplitude (circle and blue) and phase (square and yellow) hysteresis loops from the local piezoelectric response as a function of bias voltage in the thin films.


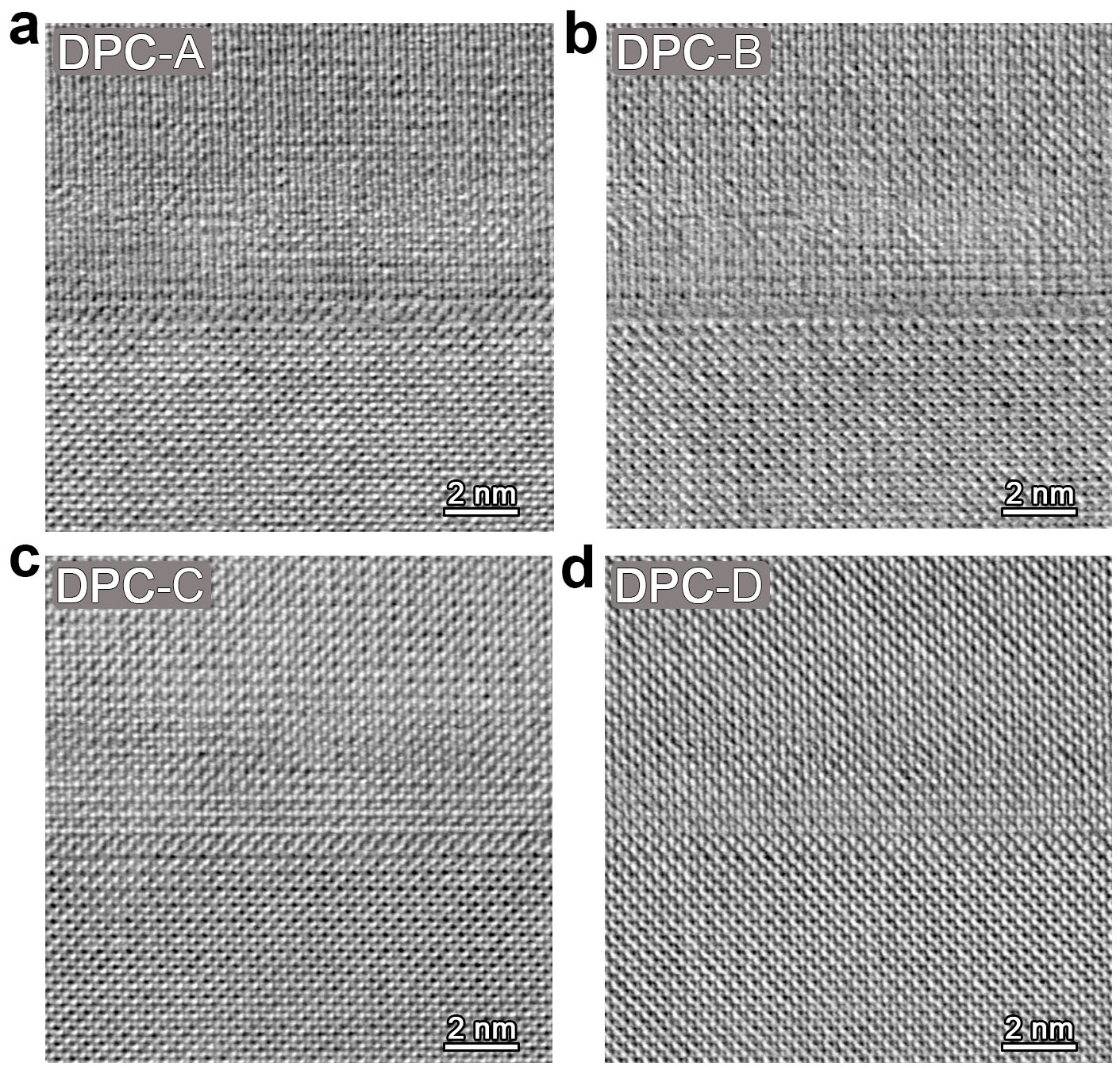


Figure S3. DPC-STEM signals for different configurations. (a) Signal A. (b) Signal B. (c) Signal C. (d) Signal D.


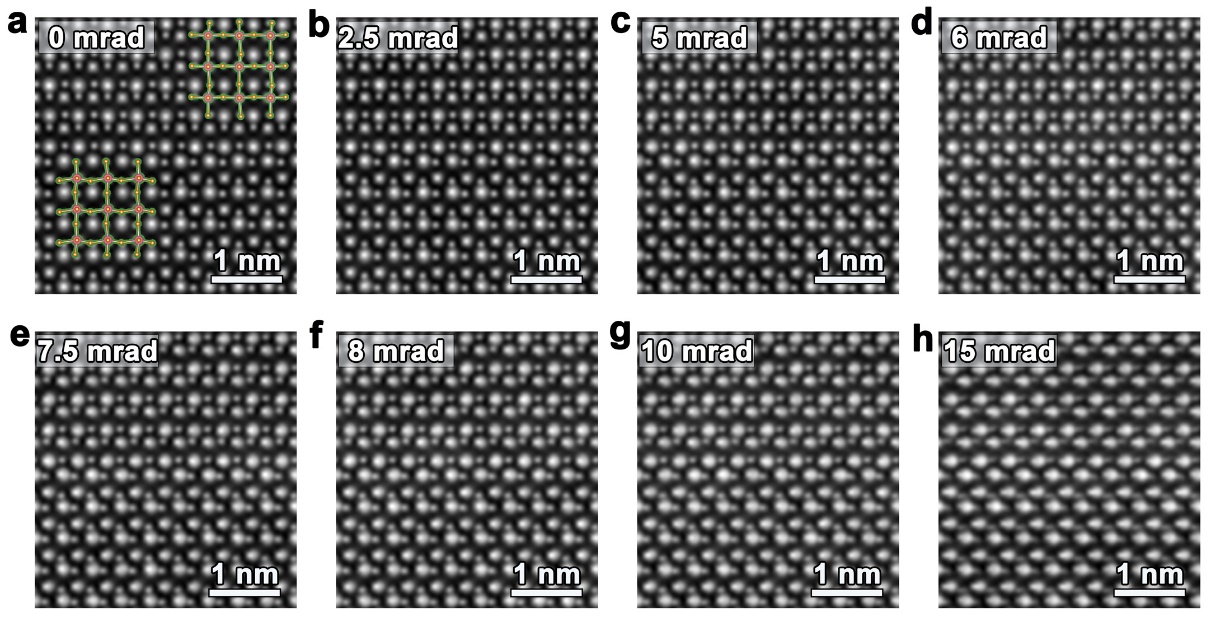


Figure S4. Simulated atomic resolution iDPC-STEM images at various sample tilt angles of (a-h) 0 mrad, 2.5 mrad, 5 mrad, 6 mrad, 7 mrad, 8 mrad, 10 mrad, 15 mrad, respectively.


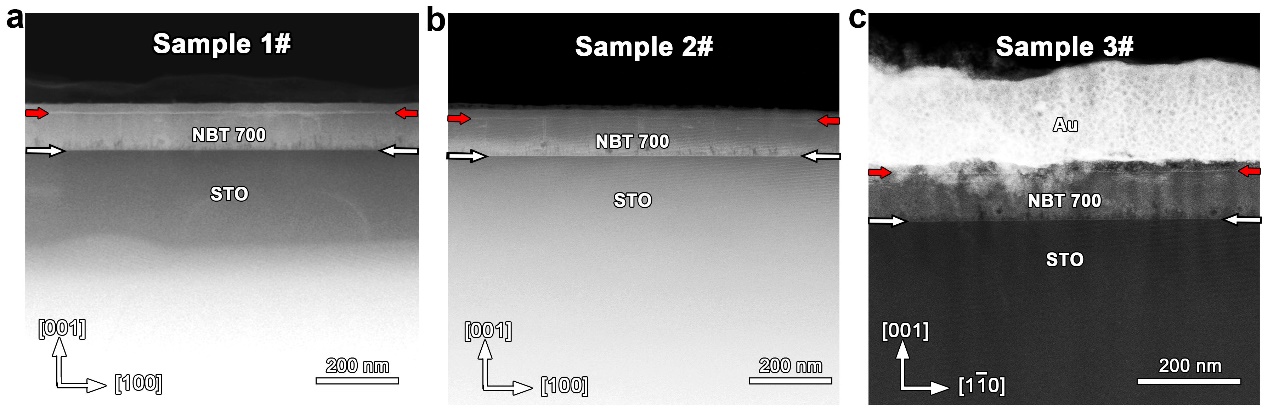


Figure S5. Cross-sectional HAADF-STEM images of NBT700 films of (a) sample 1#, (b) sample 2#, and (c) sample 3#.
